# New Stenting Technique to Achieve Favorable Jailing Configuration on Side Branch Ostium: Bent Stent Technique

**DOI:** 10.1155/2016/5198173

**Published:** 2016-03-21

**Authors:** Fumiaki Nakao

**Affiliations:** Department of Cardiology, Yamaguchi Grand Medical Center, 77 Ohsaki, Hofu, Yamaguchi 747-8511, Japan

## Abstract

According to data from stent-enhanced three-dimensional optical coherence tomography, incomplete stent apposition after side branch dilation in coronary bifurcation stenting can be reduced by the free carina type (no links bridged from a carina) and by distal cell rewiring. This is the first report to describe a bent stent technique that was devised to achieve the free carina type (no links bridged from a carina), as a favorable jailing configuration.

## 1. Introduction

Percutaneous coronary intervention (PCI) for a coronary bifurcation disease comprises 15–20% of all PCI procedures [1, 2]. A single stent technique, with stent implantation on only the main vessel crossing over a side branch (SB), is generally recommended [3]. Jailing struts or residual struts in front of an SB ostium after inadequate SB dilation may lead to adverse effects with regard to SB blood flow [4, 5]. According to data from stent-enhanced three-dimensional optical coherence tomography (3D-OCT), incomplete stent apposition after kissing balloon dilation (KBD) is reduced by the free carina type (no links bridged from a carina) and by distal cell rewiring [6]. However, it may be impossible to control the position of a link. The bent stent technique described in this report was devised to achieve the free carina type as a favorable jailing configuration.

## 2. Phantom Study

**Figure 1:**
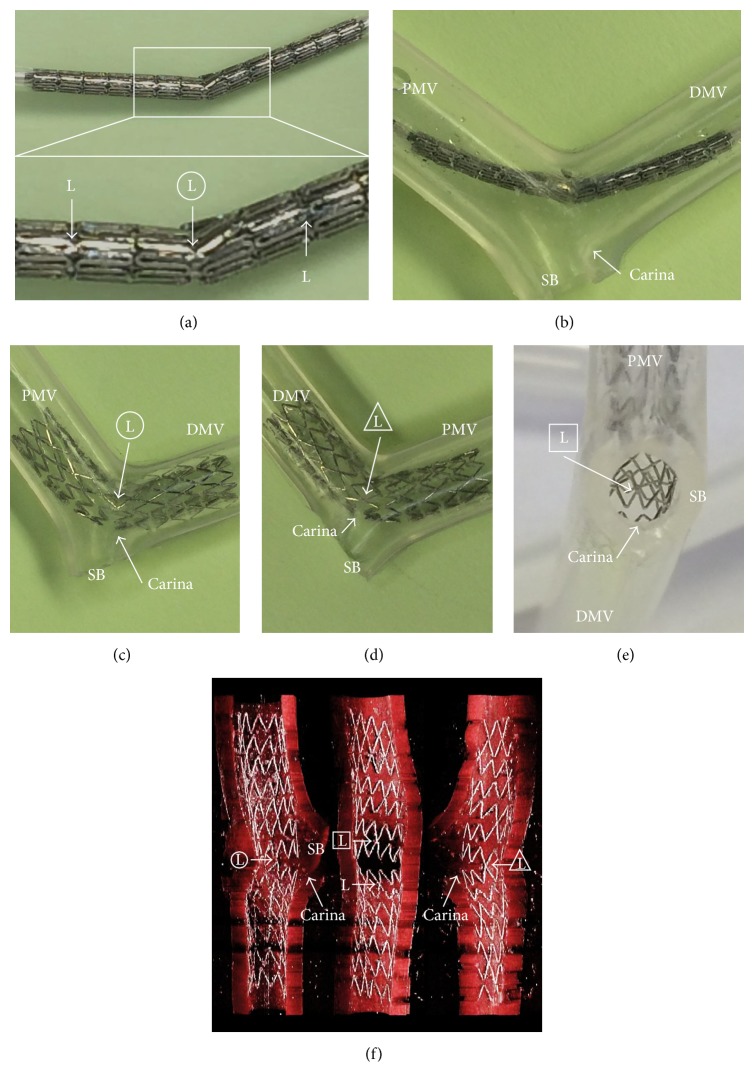
Bent stent technique in phantom study. (a) 3.5 × 18 mm two-link Biolimus-eluting stent (BES) is bent at the center articulation with laterally positioned links. (b) Bent position of this BES is matched to the carina of a phantom bifurcation vessel. After this BES is deployed, lateral view (c), opposite lateral view (d), side branch (SB) ostium from outside (e), and instant stent-accentuated three-dimensional optical coherence tomography (f) show free carina type. PMV: proximal main vessel; DMV: distal main vessel; L: link. “Ls” enclosed by circle, triangle, or square indicate the same links, respectively.

A 3.5 × 18 mm two-link Biolimus-eluting stent (BES, Nobori*™*, Terumo, Tokyo) was bent at the center articulation with laterally positioned links (Figure 1(a)), and there were no links on the greater curvature side of its bent position. There was some angle between the proximal main vessel (PMV) and the distal main vessel (DMV), and an SB is generally located on the greater curvature side in vivo. When the bent position of the bent BES was matched to the carina of a phantom bifurcation vessel along the guidewire curved by the angle between the PMV and DMV, the convex side of the bent stent was automatically positioned towards the carina (Figure 1(b)). After the bent BES was deployed, lateral-sided views (Figures 1(c) and 1(d)) with the SB ostium viewed from the outside (Figure 1(e)) and instant stent-accentuated 3D-OCT (iSA3D-OCT) that was reconstructed from OCT (Dragonfly*™* JP, St. Jude Medical, St. Paul, MN) by freeware ImageJ 1.47v (National Institute of Health, Bethesda, MD) with macroprograms of my own design [7, 8] indicated achievement of the free carina type (Figure 1(f)).

## 3. First Clinical Case

**Figure 2:**
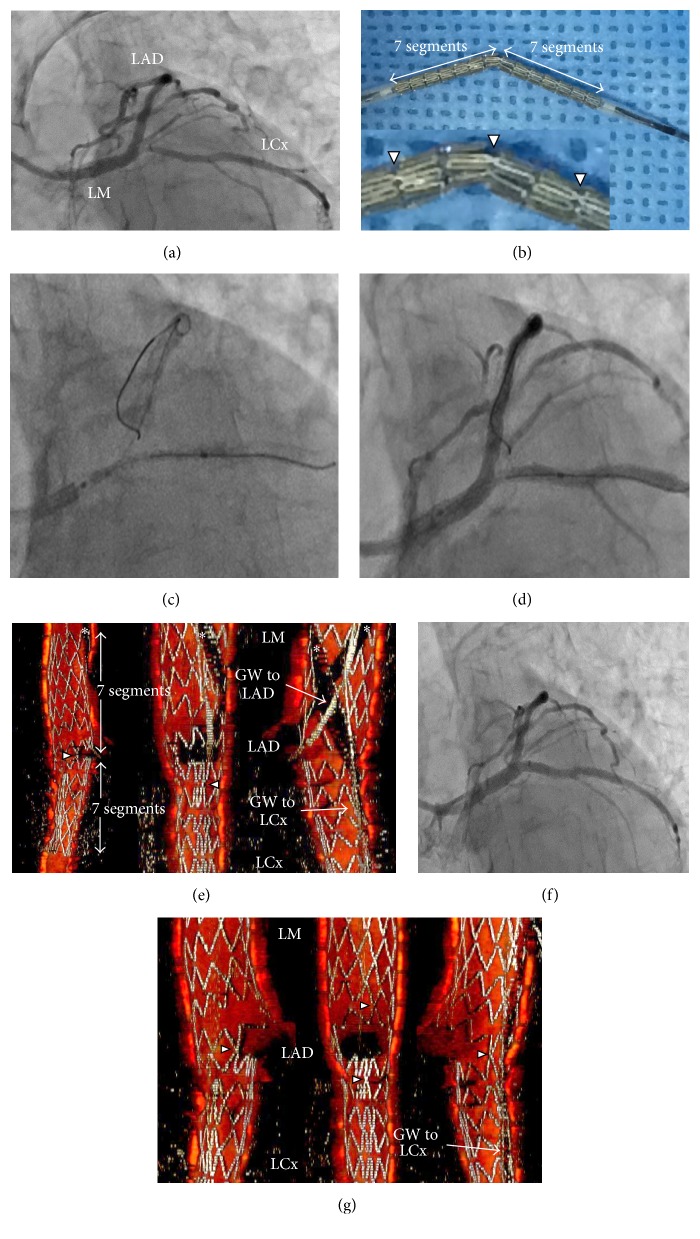
First clinical case treated with the bent stent technique. (a) Baseline coronary angiography (CAG). (b) 3.5 × 18 mm two-link Biolimus-eluting stent is bent at the center articulation with laterally positioned links. Stenting position is confirmed by X-ray fluorography (c) and CAG (d). (e) Instant stent-accentuated three-dimensional optical coherence tomography (iSA3D-OCT) shows free carina type and distal cell rewiring. After kissing balloon dilation is performed, final CAG (f) and iSA3D-OCT (g) show a good result. LM: left main coronary artery; LAD: left anterior descending artery; LCx: left circumflex artery; GW: guidewire. Arrowheads and asterisks indicate links and guidewire shadow artifacts, respectively.

Percutaneous coronary intervention was performed on a 79-year-old woman for stenosis of the proximal left circumflex artery (LCx) (Figure 2(a)). A 3.5 × 18 mm BES was bent at the center articulation with laterally positioned links (Figure 2(b)). The center of this BES was matched to the carina of the left main bifurcation (Figures 2(c) and 2(d)) and this BES was deployed on the left main coronary artery-LCx. After the guidewire was recrossed to the left anterior descending artery, the iSA3D-OCT, reconstructed from OCT (Dragonfly OPTIS*™*, St. Jude Medical) as described above, showed the free carina type and distal cell rewiring (Figure 2(e) and clip 1 in Supplementary Material available online at http://dx.doi.org/10.1155/2016/5198173). After KBD was performed, final coronary angiography (Figure 2(f)) and iSA3D-OCT (Figure 2(g) and clip 2 in Supplementary Material available online) showed a good result.

## 4. Discussion

The bent stent technique can be limited by deviation of an SB ostium from the greater curvature side of a main vessel, by positioning with X-ray fluorographic guidance, or by crossability lowered by bending. Even if an SB ostium shifts slightly from the greater curvature of a main vessel or the convex side of the bent BES shifts slightly from a carina, links of bent position may be positioned on the lateral sides of an SB ostium by the bent stent technique and therefore can be successfully apposed to a lateral wall by KBD. Precise measurement of the lesion length is required to decide the stent length and bent position. Stenting position has to be decided by X-ray fluorography and coronary angiography, and an error of the width of only one segment is allowed. In this case, the bent position was at the center, and the bent BES was therefore easily positioned. Angiography system with the stent enhancement program, which my institution does not possess, may be helpful for positioning of stents. It may not be possible to insert a bent stent to a distal lesion, because of its lowered crossability; however, the bent stent technique may be suitable for left main bifurcation stenting that requires larger dilatation of an SB and that does not require higher crossability. In culotte stenting, floating struts on an SB ostium after first-stent implantation can affect second-stent implantation and therefore adequate SB dilation after first-stent implantation is required. The bent stent technique may help to achieve the free carina type. Confirmation of the distal cell rewiring by stent-enhanced 3D-OCT is also important. I conclude that the bent stent technique has the potential to improve outcomes in patients undergoing bifurcation stenting but needs further study to indicate that.

## Supplementary Material

Clip 1 Intraprocedural instant stent-accentuated three-dimensional optical coherence tomography (iSA3D-OCT). A two-link Biolimus-eluting stent (BES), bent at the center articulation with laterally positioned links, was deployed on the left main coronary artery-left circumflex artery. After the guidewire was recrossed to the left anterior descending artery, the intraprocedural iSA3D-OCT showed the free carina type (no links bridged from a carina) and distal cell rewiring
Clip 2 Final instant stent-accentuated three-dimensional optical coherence tomography (iSA3D-OCT). After kissing balloon dilation was performed, final iSA3D-OCT showed no floating struts on side branch ostium.
